# Type 2 Diabetes Alters Vascular Cannabinoid Receptor 1 Expression, Phosphorylation Status, and Vasorelaxation in Rat Aorta

**DOI:** 10.3390/molecules25214948

**Published:** 2020-10-26

**Authors:** Enrique Sánchez-Pastor, Xóchitl Trujillo, Christian Ramos-Flores, Mónica Ríos-Silva, Felipa Andrade, Yolitzy Cárdenas, Elena Castro, Zorayda Urzúa, Oscar Newton-Sánchez, Miguel Huerta

**Affiliations:** 1 Enrico Stefani Research Unit, University Center for Biomedical Research, University of Colima, Colima CP 28045, Mexico; espastor@ucol.mx (E.S.-P.); rosio@ucol.mx (X.T.); hugo.ramosf@imss.gob.mx (C.R.-F.); mrios@ucol.mx (M.R.-S.); rosa_cardenas@ucol.mx (Y.C.); ecastro@ucol.mx (E.C.); 2 Mexican Institute of Social Security, Family Medicine Unit #168, Tepatitlan de Morelos, Jalisco CP 47600, Mexico; 3 National Technological Institute of Mexico/Technological Institute of Colima, Avenida Tecnológico No. 1, Villa de Álvarez, Colima CP 28976, Mexico; felipa.andrade@colima.tecnm.mx; 4 Mexican Institute of Social Security, Family Medicine Unit #17, Manzanillo, Colima CP 28219, Mexico; zorayda_urzua@ucol.mx; 5 Faculty of Medicine, University of Colima, Colima CP 28040, Mexico; onewton@ucol.mx

**Keywords:** cannabinoid, diabetes, CB_1_ receptor expression, vasorelaxation, CNR1

## Abstract

Previous studies have suggested a role of the endocannabinoid system in metabolic diseases, such as diabetes. We investigated the effect of diabetes on cannabinoid receptor type 1 (CB_1_) expression and cannabinoid-induced vasorelaxation in rat aorta rings. Aortas from healthy rats and from rats with experimentally induced diabetes were used to compare the vasorelaxant effect of the cannabinoid agonist arachidonylcyclopropylamide (ACPA) and CB_1_ expression and localization. After 4–8 weeks of diabetes induction, CB_1_ receptor expression and CB_1_ phosphorylation were higher in aortic rings, in association with greater vasorelaxation induced by the CB_1_ agonist ACPA compared to healthy rats. The vasorelaxant effect observed in healthy rats is similar throughout the study. Further studies are needed to elucidate the implications of CB_1_ receptor overexpression in diabetes and its influence on the progression of the cardiovascular complications of this metabolic disease.

## 1. Introduction

Type 2 diabetes mellitus is one of the most prevalent metabolic disorders worldwide. The endocannabinoid system participates in the control of energy homeostasis [1]. Previous findings indicate that the endocannabinoid system plays a critical role in gene expression of cannabinoid receptor type 1 (CB_1_; also known as CNR1) in the β pancreatic islets and other tissues [2], suggesting a role of the endocannabinoid system in some metabolic diseases, such as type 2 diabetes mellitus. Thus, substances that activate or antagonize CB_1_ could also affect diabetes endpoints. In addition, elevated glucose increases CB_1_ expression in the kidney, pancreas, subcutaneous adipose tissue and nervous system [2,3,4,5]. Regarding cardiovascular effects, the CB_1_ and cannabinoid receptor type 2 (CB_2_) agonist WIN 55,212-2 elicits vasorelaxation in rat aorta [6]. However, the effect of the activation of these receptors on type 2 diabetes mellitus has not been elucidated. As we reported recently [7], pre-incubation of rat aortic rings with the CB_1_ agonist arachidonylcyclopropylamide (ACPA) results in vasorelaxation. This vasorelaxant effect is completely blocked by the CB_1_ antagonist AM281, suggesting a role of CB_1_ activation in the regulation of vascular tone.

Taken together, these findings suggest a dual role of CB_1_ in both glucose regulation and vaso-responsiveness, depending on the activation or inhibition of the receptor. The aim of the present study was to evaluate whether diabetes alters the vasorelaxant effects of the CB_1_ agonist ACPA in aortic rings and CB_1_ receptor expression or phosphorylation in the aorta.

## 2. Results

### 2.1. Body Weight and Serum Glucose

Bodyweight and fasting glucose were measured before and at 2, 4, and 8 weeks after the initiation of experimental diabetes (Table 1). After 4 and 8 weeks, the weight of the diabetic rats was decreased compared to their initial weight; diabetic rats had elevated fasting glucose levels compared to their initial levels and with respect to control rats (Table 1).

### 2.2. Effects of Cannabinoids on Vasorelaxation of Aortic Rings from Diabetic and Healthy Rats

**Figure 1:**
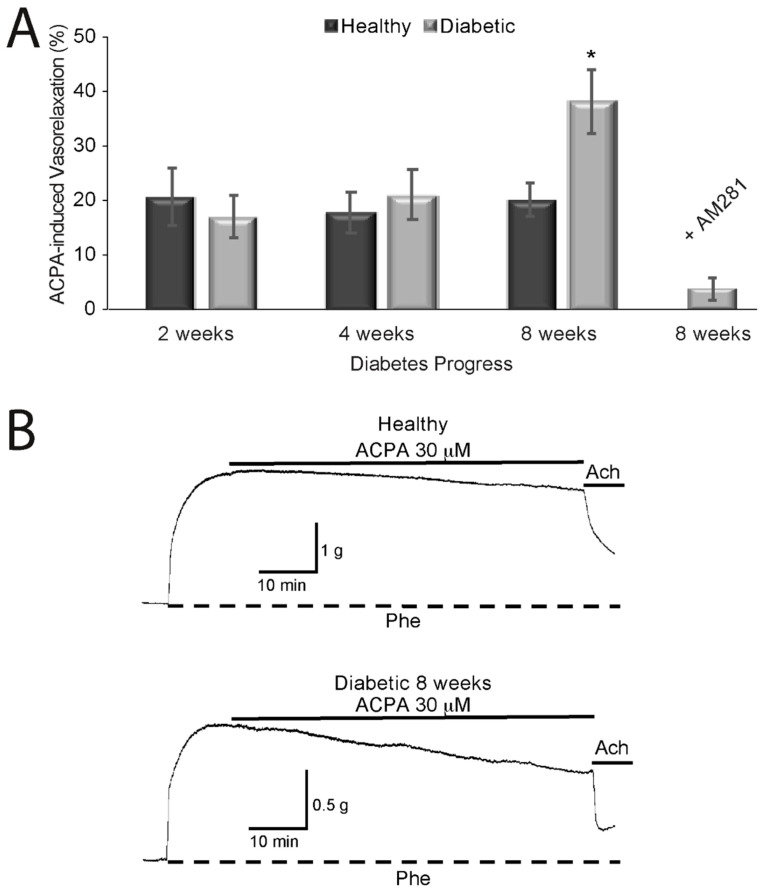
Vasorelaxant effect of arachidonylcyclopropylamide (ACPA) on healthy and diabetic rat aortic rings. (**A**). Summary of the vasorelaxant effect induced by ACPA at different times after diabetes induction. Dark bars correspond to healthy rats, and gray bars correspond to diabetic rats after 2, 4 or 8 weeks after Streptozotocin administration. The effect of ACPA when cannabinoid receptor type 1 (CB_1_) receptors were blocked with AM281 is shown in the last gray bar. * Significant (*p* < 0.05) change in tension compared to Phe precontraction. (**B**) Isometric tension recording of phenylephrine (Phe)-induced contraction of aortic rings. The top lines indicate exposure to ACPA. Upper trace: vasorelaxant effect of ACPA on a healthy ring. Lower trace: effect of ACPA on a diabetic ring. The relaxation of tension was measured at 60 min of ACPA treatment. The vasorelaxant effect was more pronounced in aortic rings from diabetic rats than those from healthy rats (*n* = 5 rats).

We previously reported that ACPA causes vasorelaxation of aortic rings from healthy rats through activation of CB_1_, an effect that is blocked when the rings are pre-incubated with the CB_1_ antagonist AM281 [7]. To explore the effects of CB_1_ activation on the vascular tone of aortic rings during the progression of diabetes in rats (2, 4, and 8 weeks), we used ACPA, a selective agonist for CB_1_. ACPA induced vasorelaxation, reaching a maximum of 20.64 ± 5.2% (*n* = 4 rats) and 16.94 ± 3.84% (*n* = 4 rats) at two weeks; 17.74 ± 3.83% (*n* = 5 rats) and 20.98 ± 4.56% (*n* = 5 rats) at four weeks; and 20.17 ± 3.04% (*n* = 7 rats, *p* < 0.01) and 37.87 ± 5.63% at eight weeks (*n* = 7 rats, *p* < 0.001 relative to the control condition; Figure 1A) for healthy and diabetics rats, respectively. We reported previously and corroborated here that the maximum vasorelaxation induced by the same concentration of ACPA in aortic rings from healthy rats was about 20% throughout the study. Thus, the vasorelaxant effect of the cannabinoid ACPA on aortic rings from diabetic rats in the present study was more pronounced than the slower vasorelaxation effect of ACPA on aortic rings from healthy rats in the previous study. Furthermore, the vasorelaxant effect of ACPA on aortic rings from diabetic rats after eight weeks of diabetes induction was almost completely reversed when the rings were incubated with the CB_1_ antagonist AM281 30 min previous to the addition of ACPA (3.86 ± 1.78%; *n* = 5 rats). Figure 1B shows contractures by phenylephrine and the effect of ACPA on healthy (upper trace) and diabetic rats (lower trace). ACPA induced a more pronounced relaxation in aortic rings from diabetic rats than those from healthy rats. A smaller contracture induced by 1 μM Phe was observed in aortic rings from diabetic rats as above described.

### 2.3. CB_1_ Receptor Expression, Phosphorylation Status and Localization in Diabetic Rat Aorta

**Figure 2:**
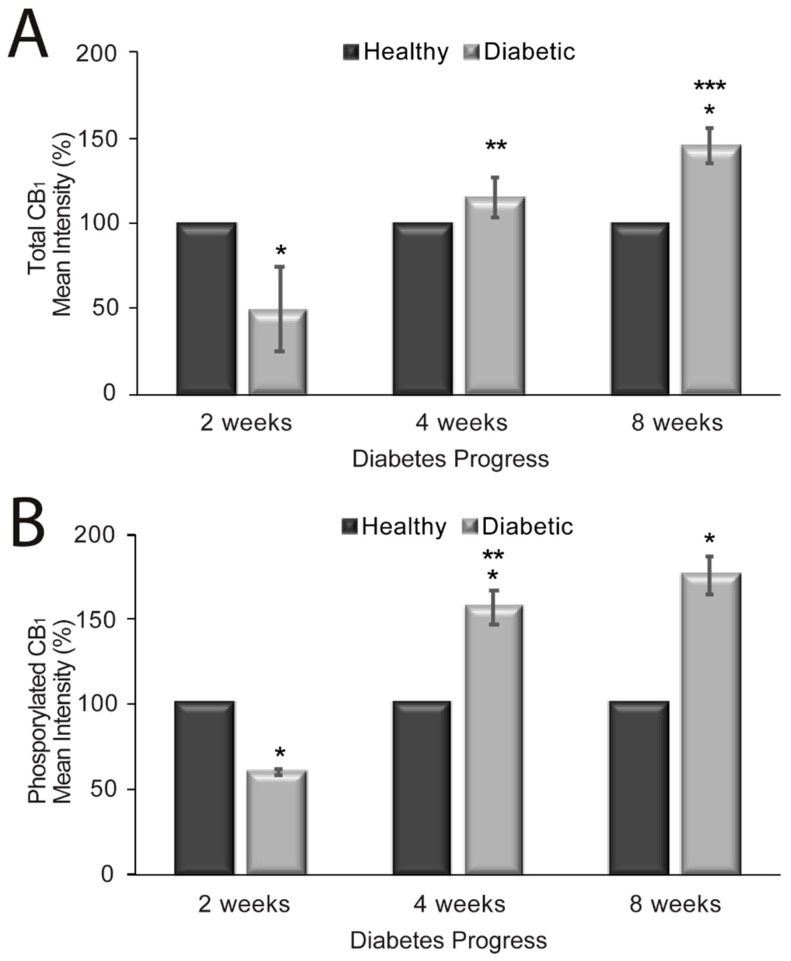
Mean intensity of total CB_1_ receptors (**A**) and phosphorylated CB_1_ receptors (**B**) in healthy and diabetic rat aortas. *n* = 10 image stacks for each. * Significant (*p* < 0.05) change in CB_1_ mean intensity in diabetic vs. healthy aortic rings at different time points. ** Significant difference (*p* < 0.05) in CB_1_ mean intensity between 2 and 4 weeks. *** Significant difference (*p* < 0.05) in CB_1_ mean intensity between 4 and 8 weeks.

To further explore the role of CB_1_ receptors in diabetes, we explored whether the increased vasorelaxant effect seen in diabetes is related to a modulation of CB_1_ expression in aortic rings from diabetic rats and/or a change in the phosphorylation status of the receptor. These issues were addressed in immunohistochemistry experiments with an anti-CB_1_ antibody that labels total CB_1_ receptors or by labeling only phosphorylated CB_1_ receptors. The experiments were performed in parallel in aortic rings from healthy and diabetic rats using the same amounts of antibodies and incubation times. Confocal images were acquired using the same parameters. The mean immunofluorescence intensities of total CB_1_ and phosphorylated CB_1_ were analyzed at distinct times (2, 4, and 8 weeks) of diabetes progression vs. controls (i.e., healthy rats) as shown in Figure 2. Total CB_1_ receptors had a mean intensity of 100 ± 8.03% and 49.85 ± 24.72% (*p* = 0.04; *n* = 4 rats) after 2 weeks; 100 ± 4.37% and 115.68 ± 11.84% (*p* = 0.31; *n* = 4 rats) after 4 weeks; and 100 ± 6.29% and 145.83 ± 10.14% (*p* < 0.02; *n* = 4 rats) at 8 weeks for healthy and diabetic rats, respectively. Thus, total CB_1_ receptor expression is increased in the rat aorta after eight weeks of diabetes induction (Figure 2A). Phosphorylated CB_1_ receptors had a mean intensity of 100 ± 5.12% and 60.29 ± 2.04% (*p* < 0.0001; *n* = 4 rats) at 2 weeks; 100 ± 7.07% and 157.23 ± 9.89% (*p* = 0.006; *n* = 4 rats) at 4 weeks; and 100 ± 11.06% and 176.59 ± 11.20% (*p* = 0.01; *n* = 4 rats) at 8 weeks for healthy and diabetic rats, respectively (Figure 2B). A significant increase in the mean intensity of phosphorylated CB_1_ was observed after four weeks of diabetes progression (2 vs. 4 weeks, *p* < 0.001; *n* = 4 rats). The immunofluorescence did not differ significantly between 4 and 8 weeks.

**Figure 3:**
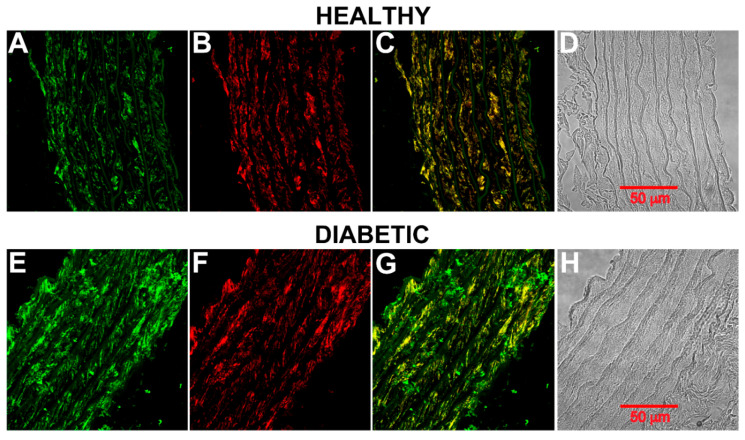
Phosphorylated CB_1_ receptor on rat aortic smooth muscle. (**A**) Healthy rat aortic ring showing CB_1_ receptor (green) and (**B**) smooth muscle α-actin (red). (**C**) An overlay of (**A**) and (**B**). (**D**) Bright-field microscopy image of the aortic ring. (**E**) Diabetic rat aortic ring (8 weeks) showing CB_1_ receptor (green) and (**F**) smooth muscle α-actin (red). (**G**) An overlay of (**E**) and (**F**). (**H**) Bright-field microscopy image of the aortic ring.

Figure 3 shows phosphorylated CB_1_ receptor labeling in aortas from healthy and diabetic rats eight weeks after diabetes onset. CB_1_ is localized in smooth muscle (Figure 3B,C,F,G). The intensity of the signal was higher in diabetic rings than in healthy rings (compare Figure 3A,E). Similar results were obtained when total CB_1_ receptors were labeled.

## 3. Discussion

The role of the endocannabinoid system has recently emerged as being important in the pathogenesis of type 2 diabetes mellitus, which is a well-known risk factor for cardiovascular disease and heart failure, though the mechanisms involved are not well-understood [8,9,10,11].

Diabetes has been shown to decrease the maximum relaxation and sensitivity to Acetylcholine, with hyperglycemia being the major causal factor in the development of this endothelial dysfunction [12]. Delta-9-tetrahydrocannabinol treatment and some endocannabinoids improve endothelium-dependent relaxation of the aorta in the Zucker rat model of type 2 diabetes, as well as the STZ/nicotinamide-induced diabetic rat model [12,13,14,15]. However, vascular endothelium-independent mechanisms have not been studied. Here, we showed that ACPA has an increased vasorelaxant effect on the aorta by direct modulation of CB_1_ receptors on the artery smooth muscle cells [7].

The aim of the present study was to evaluate the possible changes in the expression of CB_1_ receptors on thoracic aorta from streptozotocin-induced diabetic rats and its functional role by investigating the in vitro effects of the administration of a cannabinoid CB_1_ receptor agonist on aortic rings. Recently, evidence has accumulated regarding the vasorelaxant effects of cannabinoids in isolated blood vessel preparations [8,9]. Several studies have reported hypotensive effects of the endocannabinoid anandamide [13,14], and an enhancement of these effects has been shown in hypertensive rats [16,17,18,19], as well as increased circulating levels of this endocannabinoid in diabetic patients [20], suggesting that anandamide could have beneficial vascular effects. In 2012, Hopps et al. [21] reported enhanced vasorelaxant effects of oleamide in hypertension and that this increase could be explained by an adaptation to increased blood pressure. However, there have been no reports of the effects of cannabinoids in diabetes. The effects of anandamide reported in these studies were mediated by the activation of both CB_1_ and TRPV1 receptors. Thus, we used a potent and selective CB_1_ receptor agonist to evaluate the effect of the activation of these receptors on the vascular tone of aortas from diabetic rats. We recently reported that the CB_1_ receptor agonist ACPA induces vasorelaxation of aortic rings from healthy rats, and this effect was mediated by the activation of CB_1_ receptors, BK_Ca_^2+^ channels, and the inhibition of voltage-gated Ca^2+^ channels located at the membrane of smooth muscle cells in these arteries [7]. Our results indicate that ACPA has a more pronounced (~2 times) vasorelaxant effect on aortic rings from diabetic rats at eight weeks than the effect observed in healthy rats, which is consistent with the enhanced effects reported in hypertension. Furthermore, this vasorelaxant effect was completely blocked when a CB_1_ antagonist was used prior to the addition of ACPA, indicating that the vasorelaxant effect relies on the activation of CB_1_ receptors suggesting a mechanism similar to that reported for healthy rats [7].

The modulation of CB_1_ receptors has already been reported in some diseases. During hyperglycemia, the expression of CB_1_ receptors in the frontal cortex of mice is reduced [22], but they are overexpressed in the kidney, pancreas and nervous system [2,4,5]. In addition, CB_1_ receptors are overexpressed in the vasculature of hypertensive rats, which is related to the increased vasorelaxant effect caused by cannabinoids [13,19]. Therefore, in order to evaluate the possible changes in CB_1_ expression in the aortas of diabetic rats, we analyzed total CB_1_ receptor expression and phosphorylated CB_1_ receptors by confocal microscopy. Our results show that CB_1_ receptors are expressed at higher levels on aortic rings from diabetic rats compared to those from healthy rats. Thus, the enhanced vasorelaxant effect induced by ACPA in diabetic rats could be explained by the overexpression of CB_1_ receptors on aortic smooth muscle cells. These findings agree with previous studies in other tissues [2]. The vasorelaxant effect increases after eight weeks of diabetes induction, which is consistent with CB_1_ receptor overexpression after eight weeks. This overexpression could be relevant in other intracellular signaling pathways, as CB_1_ activation has also been reported to contribute to vascular inflammation [23,24], induce cell death in human coronary artery endothelial cells [25] and promote atherosclerosis [26]. On the other hand, CB_1_ receptor phosphorylation at serine 316 has been reported to reduce the receptor effects [5,27]. This increase in phosphorylated CB_1_ receptors could represent a compensatory mechanism for the increase in total CB_1_ receptor expression. Although phosphorylated CB_1_ receptors increase from week 4 to 8, a significant increase in the vasorelaxant effect of ACPA on aortic rings from diabetic rats was observed at eight weeks, indicating that this compensatory response did not affect the activation of CB_1_ receptors in our study.

Our results also show that aortic rings from diabetic rats have decreased reactivity to phenylephrine, which was determined previously in other studies [28] and may be related to a decrease in the expression of α-adrenergic receptors associated with high glucosuria [29].

In our earlier study, we showed that ACPA activation of CB_1_ in smooth muscle results in vasorelaxation of aortic rings in healthy rats [7], and in the current study, we link diabetes to greater vasorelaxation of aortic rings. Thus, the current findings add to a suggestive picture of the interaction between diabetes, CB_1_ and cardiovascular responses, suggesting that upregulation of the endocannabinoid system may positively alter vascular function. Previous studies have suggested that activation of CB_1_ is involved in the development of cardiovascular complications of diabetes [10]. Our results suggest that diabetes per se may increase total CB_1_ receptor expression and phosphorylated CB_1_ receptors in aortic rings and indicate a potential target for vascular complications in experimental diabetes [12,30], as it has been proposed for other disorders associated with type 2 diabetes mellitus [31]. Recent studies have shown the potential therapeutic role of the endocannabinoid system in diabetes complications, including the treatment of diabetic nephropathy [32].

Further studies are needed to elucidate other possible implications of CB_1_ receptor overexpression in the aorta in diabetes and its influence on the progression of the cardiovascular complications of this metabolic disease.

## 4. Materials and Methods

### 4.1. Animals

Male Wistar rats (approximately 2 months old, weight 230–350 g) were randomly divided into groups, one of healthy rats and one to undergo experimentally induced diabetes. All animals were maintained under the same conditions at the laboratory animal facility of the Centro Universitario de Investigaciones Biomédicas at Universidad de Colima, México. The rats were housed under standard light/dark conditions (12 h light/12 h dark) at 22 ± 2 °C. They were fed ad libitum with water and rodent food (Envigo Labs Corporation, Indianapolis, IN, USA). We conducted all experiments and animal management protocols in accordance with the ethical standards of the Mexican Official Norm technical specifications for the production, care and use of laboratory animals (NOM-062-ZOO-1999) and recommendations listed in the Guide for the Care and Use of Laboratory Animals from the United States National Institutes of Health.

### 4.2. Experimental Induction of Diabetes

We induced diabetes in rats by a single intraperitoneal injection of 45 mg of STZ (Sigma-Aldrich, St. Louis, MO, USA) per kilogram of body weight [33]. STZ damages the pancreatic β cells, affecting serum glucose. Fasting blood glucose ≥200 mg/dL was used to confirm a diabetic state [33].

### 4.3. Cannabinoid Administration

ACPA (Tocris, Bristol, UK) was dissolved in Tocrisolve and used at 30 µM as described in a previous in vitro study [7].

### 4.4. Glucose Measurements

We measured glucose parameters after 12 h of overnight fasting, as described previously [34].

### 4.5. Removal and Preparation of Aortas

Diabetic and healthy male Wistar rats were anesthetized and euthanized by intraperitoneal administration of pentobarbital. The aorta was removed via an incision in the thoracic cavity and placed on a Petri dish with Krebs–Henseleit solution (118 mM NaCl, 5 mM KCl, 1.2 mM MgSO_4_, 1.2 mM KH_2_PO_4_, 25 mM NaHCO_3_, 2 mM CaCl_2_, 2 g of D-glucose; pH 7.4). Aortas were fixed with 4% paraformaldehyde for 24 h for immunohistochemical experiments or cut into 3 µm rings for tension recordings.

### 4.6. Aortic Ring Tension Recordings

Aortic rings were placed in a 10 mL tissue bath filled with Krebs–Henseleit solution, bubbled with 95% O_2_ and 5% CO_2_ and maintained at 37 °C in a circulating water bath. The rings were placed between two stainless steel wires; one wire was fixed to a manipulator, and the other was connected to an isometric force transducer (Radnoti Glass Technology Inc., Monrovia, CA, USA). The force transducer was connected to a CyberAmp (Axon Instruments, Foster City, CA, USA) and the signal acquired with a Digidata 1200 (Axon Instruments, Foster City, CA, USA) in the Axoscope subroutine of pClamp (version 9; Axon Instruments, Foster City, CA, USA). Aortic rings were precontracted with 1 µM phenylephrine and, when contraction was stable (this was set at 100%), we added 30 µM ACPA to the bath. We performed analyses using the Clampfit subroutine. The experiments were performed in duplicate, and the data averaged. Results are expressed as the mean ± standard error of the measurements from at least 8 aorta rings from at least four different rats.

### 4.7. Immunohistochemical Analysis of Aortic Rings

After fixation in paraformaldehyde, the aorta was embedded in paraffin and cut into 3-µm aortic rings, which were placed on glass slides. The slides were washed three times with xylene for 10 min each (Sigma-Aldrich, St. Louis, MO, USA). We then rehydrated the rings by washing for 3 min with decreasing concentrations of ethanol (95%, 70%, 50% and 30%; three times each). Next, the rings were incubated in 10% normal goat serum in phosphate-buffered saline (PBS)/0.2% Triton X-100 for 30 min at room temperature to block background staining and permeabilize the cells. After being blocked, the rings were incubated overnight at 4 °C with anticannabinoid receptor I antibody (1:100; cat. no. ab23703, Abcam, Cambridge, MA, USA) or recombinant anticannabinoid receptor I (phospho S316) antibody [EPR2223(N)] (1:100; cat. no. ab186428, Abcam, Cambridge, MA, USA) and anti-alpha smooth muscle actin [1A4] antibody (1:50; cat. no. ab7817, Abcam, Cambridge, MA, USA). The next day, we washed the slides in PBS with 0.2% Triton X-100 and 1% normal goat serum three times for 5 min each. Secondary antibodies (1:100 FITC-conjugated anti-rabbit antibody (1:100; cat. no. ab6717, Abcam, Cambridge, MA, USA) and 1:50 goat anti-mouse IgG (H+L) cross-adsorbed secondary antibody, Alexa Fluor 568 (cat. no. A-11004, Thermo Fisher Scientific, Waltham, MA, USA) were added to the aortic rings, which we then incubated for 1 h in the dark at room temperature. Finally, we washed the slides three times in PBS and mounted coverslips using ProLong Gold Antifade reagent with DAPI (Thermo Fisher Scientific, Waltham, MA, USA). The specificity of the antibodies was evaluated by performing experiments in the absence of the first antibodies and by pre-absorbing the cannabinoid receptor I antibody with its blocking peptide (cat. # ab50542, Abcam, Cambridge, MA, USA) 30 min prior to addition to the samples.

We acquired confocal images using an LSM700 Zeiss confocal microscope using a 40× objective with a numeric aperture of 1.3. The mean intensity of the immunofluorescence of 10 different images from four different experiments was calculated using ImageJ software (National Institutes of Health, Bethesda, MD, USA) [35].

### 4.8. Statistical Analysis

We performed descriptive statistical analyses using Stata software (version 11). Variables are reported as mean and standard error. We used paired Student’s *t*-tests to assess differences in mean values at the beginning and end of each intervention and Student’s *t*-tests for independent samples to assess differences in the mean values recorded for rats with experimentally induced diabetes vs. control rats. Analysis of variance (ANOVA) was used to assess differences between groups. Significance was considered when *p* < 0.05.

## Figures and Tables

**Table 1:** Bodyweight and serum glucose levels in rats.

	2 Weeks		4 Weeks		8 Weeks	
	Initial	Final	Initial	Final	Initial	Final
*Body Weight (g)*						
Healthy	279 ± 3.84	322 ± 8.23 *	319 ± 14.4	338 ± 4.85	321 ± 6.69	342 ± 8.71 *
Diabetes	235 ± 9.3	263 ± 10.8 *	280 ± 8.16	243 ± 9.9	308 ± 13.33	275 ± 14.65
*Fasting Glucose (mg/dL)*						
Healthy	81 ± 1.78	85 ± 2.56	76.80 ± 2.2	78 ± 2.21	83 ± 4.49	77 ± 1.5
Diabetes	319 ± 19.28	405 ± 19.83 *	288 ± 18.46	478 ± 34.7 *	310 ± 13.13	441 ± 13.71 *

Data are presented as mean ± standard error. * *p* < 0.05 Student’s *t*-test for paired samples.

## References

[1] Cristino L., Becker T., Di Marzo V. (2014). Endocannabinoids and energy homeostasis: An update. BioFactors.

[2] Vilches-Flores A., Delgado-Buenrostro N.L., Navarrete-Vázquez G., Villalobos-Molina R. (2010). CB1 cannabinoid receptor expression is regulated by glucose and feeding in rat pancreatic islets. Regul. Pept..

[3] Sidibeh C.O., Pereira M.J., Lau Börjesson J., Kamble P.G., Skrtic S., Katsogiannos P., Sundbom M., Svensson M.K., Eriksson J.W. (2017). Role of cannabinoid receptor 1 in human adipose tissue for lipolysis regulation and insulin resistance. Endocrine.

[4] Jenkin K.A., Mcainch A.J., Zhang Y., Kelly D.J., Hryciw D.H. (2015). Elevated cannabinoid receptor 1 and G protein-coupled receptor 55 expression in proximal tubule cells and whole kidney exposed to diabetic conditions. Clin. Exp. Pharmacol. Physiol..

[5] Díaz-Asensio C., Setién R., Echevarría E., Casis L., Casis E., Garrido A., Casis O. (2008). Type 1 diabetes alters brain cannabinoid receptor expression and phosphorylation status in rats. Horm. Metab. Res..

[6] Dannert M.T., Alsasua A., Herradon E., Martín M.I., López-Miranda V. (2007). Vasorelaxant effect of Win 55,212-2 in rat aorta: New mechanisms involved. Vascul. Pharmacol..

[7] Sánchez-Pastor E., Andrade F., Sánchez-Pastor J.M., Elizalde A., Huerta M., Virgen-Ortiz A., Trujillo X., Rodríguez-Hernández A. (2014). Cannabinoid receptor type 1 activation by arachidonylcyclopropylamide in rat aortic rings causes vasorelaxation involving calcium-activated potassium channel subunit alpha-1 and calcium channel, voltage-dependent, L type, alpha 1C subunit. Eur. J. Pharmacol..

[8] Hillard C.J. (2000). Endocannabinoids and vascular function. J. Pharmacol. Exp. Ther..

[9] Randall M.D., Kendall D.A., O’Sullivan S. (2004). The complexities of the cardiovascular actions of cannabinoids. Br. J. Pharmacol..

[10] Gruden G., Barutta F., Kunos G., Pacher P. (2016). Role of the endocannabinoid system in diabetes and diabetic complications. Br. J. Pharmacol..

[11] Horvth B., Mukhopadhyay P., Hask G., Pacher P. (2012). The endocannabinoid system and plant-derived cannabinoids in diabetes and diabetic complications. Am. J. Pathol..

[12] Altinok A., Coşkun Z.M., Karaoʇlu K., Bolkent S., Akkan A.G., Özyazgan S. (2015). Δ9-tetrahydrocannabinol treatment improved endothelium-dependent relaxation on streptozotocin/nicotinamide-induced diabetic rat aorta. Acta Physiol. Hung..

[13] Bátkai S., Pacher P., Osei-Hyiaman D., Radaeva S., Liu J., Harvey-White J., Offertáler L., Mackie K., Rudd M.A., Bukoski R.D. (2004). Endocannabinoids acting at cannabinoid-1 receptors regulate cardiovascular function in hypertension. Circulation.

[14] Wang Y., Kaminski N.E., Wang D.H. (2005). VR1-mediated depressor effects during high-salt intake: Role of anandamide. Hypertension.

[15] Stanley C.P., Wheal A.J., Randall M.D., O’Sullivan S.E. (2013). Cannabinoids alter endothelial function in the Zucker rat model of type 2 diabetes. Eur. J. Pharmacol..

[16] Li J., Kaminski N.E., Wang D.H. (2003). Anandamide-induced depressor effect in spontaneously hypertensive rats: Role of the vanilloid receptor. Hypertension.

[17] Wheal A.J., Bennett T., Randall M.D., Gardiner S.M. (2007). Cardiovascular effects of cannabinoids in conscious spontaneously hypertensive rats. Br. J. Pharmacol..

[18] Wheal A.J., Randall M.D. (2009). Effects of hypertension on vasorelaxation to endocannabinoids in vitro. Eur. J. Pharmacol..

[19] Guo Z., Liu Y.X., Yuan F., Ma H.J., Maslov L., Zhang Y. (2015). Enhanced vasorelaxation effect of endogenous anandamide on thoracic aorta in renal vascular hypertension rats. Clin. Exp. Pharmacol. Physiol..

[20] Matias I., Gonthier M.P., Orlando P., Martiadis V., De Petrocellis L., Cervino C., Petrosino S., Hoareau L., Festy F., Pasquali R. (2006). Regulation, function, and dysregulation of endocannabinoids in models of adipose and β-pancreatic cells and in obesity and hyperglycemia. J. Clin. Endocrinol. Metab..

[21] Hopps J.J., Dunn W.R., Randall M.D. (2012). Enhanced vasorelaxant effects of the endocannabinoid-like mediator, oleamide, in hypertension. Eur. J. Pharmacol..

[22] Moura L.I.F., Lemos C., Ledent C., Carvalho E., Köfalvi A. (2019). Chronic insulinopenia/hyperglycemia decreases cannabinoid CB 1 receptor density and impairs glucose uptake in the mouse forebrain. Brain Res. Bull..

[23] El-Remessy A.B., Rajesh M., Mukhopadhyay P., Horváth B., Patel V., Al-Gayyar M.M.H., Pillai B.A., Pacher P. (2011). Cannabinoid 1 receptor activation contributes to vascular inflammation and cell death in a mouse model of diabetic retinopathy and a human retinal cell line. Diabetologia..

[24] Gestrich C., Duerr G.D., Heinemann J.C., Meertz A., Probst C., Roell W., Schiller W., Zimmer A., Bindila L., Lutz B. (2015). Activation of endocannabinoid system is associated with persistent inflammation in human aortic aneurysm. BioMed Res. Int..

[25] Rajesh M., Mukhopadhyay P., Haskó G., Liaudet L., MacKie K., Pacher P. (2010). Cannabinoid-1 receptor activation induces reactive oxygen species-dependent and -independent mitogen-activated protein kinase activation and cell death in human coronary artery endothelial cells. Br. J. Pharmacol..

[26] Guillamat-Prats R., Rami M., Herzig S., Steffens S. (2019). Endocannabinoid signalling in atherosclerosis and related metabolic complications. Thromb. Haemost..

[27] Garcia D.E., Brown S., Hille B., Mackie K. (1998). Protein kinase C disrupts cannabinoid actions by phosphorylation of the CB1 cannabinoid receptor. J. Neurosci..

[28] Saleh D.O., Bayoumi A.R., El-Eraky W.I., El-Khatib A.S. (2013). Streptozotocin-induced vascular and biochemical changes in rats: Effects of rosiglitazone vs. metformin. Bull. Fac. Pharm..

[29] Bucci M., Roviezzo F., Brancaleone V., Lin M.I., Di Lorenzo A., Cicala C., Pinto A., Sessa W.C., Farneti S., Fiorucci S. (2004). Diabetic mouse angiopathy is linked to progressive sympathetic receptor deletion coupled to an enhanced caveolin-1 expression. Arterioscler. Thromb. Vasc. Biol..

[30] Vella R.K., Jackson D.J., Fenning A.S. (2017). Δ9-tetrahydrocannabinol prevents cardiovascular dysfunction in STZ-diabetic Wistar-Kyoto rats. BioMed Res. Int..

[31] Nagappan A., Shin J., Jung M.H. (2019). Role of cannabinoid receptor type 1 in insulin resistance and its biological implications. Int. J. Mol. Sci..

[32] Barutta F., Grimaldi S., Gambino R., Vemuri K., Makriyannis A., Annaratone L., Di Marzo V., Bruno G., Gruden G. (2017). Dual therapy targeting the endocannabinoid system prevents experimental diabetic nephropathy. Nephrol. Dial. Transplant..

[33] Márquez-Ibarra A., Huerta M., Villalpando-Hernández S., Ríos-Silva M., Díz-Reval M.I., Cruzblanca H., Mancilla E., Trujillo X. (2016). The effects of dietary iron and capsaicin on hemoglobin, blood glucose, insulin tolerance, cholesterol, and triglycerides, in Healthy and diabetic Wistar rats. PLoS ONE.

[34] Miron V.R., Bauermann L., Morsch A.L.B., Zanin R.F., Corrêa M., Silva A.C., Mazzanti C., Morsch V.M., Lunkes G.I., Schetinger M.R.C. (2007). Enhanced NTPDase and 5′-nucleotidase activities in diabetes mellitus and iron-overload model. Mol. Cell. Biochem..

[35] Abràmoff M.D., Magalhães P.J., Ram S.J. (2004). Image processing with image. J. Biophotonics Int..

